# Oxalate nephropathy in an elderly patient with newly diagnosed celiac disease – a case report

**DOI:** 10.1186/s12882-023-03241-y

**Published:** 2023-06-27

**Authors:** Hendrik W. Zijlstra, Coen A. Stegeman

**Affiliations:** 1 Department of critical care, University Medical Center Groningen, Groningen, The Netherlands; 2 Department of nephrology, University Medical Center Groningen, Groningen, The Netherlands

**Keywords:** Oxalate, Nephropathy, Celiac disease

## Abstract

Oxalate nephropathy, due to secondary hyperoxaluria has widely been described in gastrointestinal diseases. However, reports of oxalate nephropathy in newly diagnosed celiac disease are rare.

A 72-year-old Caucasian male presented to the hospital with abdominal discomfort and acute renal insufficiency with a creatinine of 290 µmol/L.

The clinical course, laboratory results and urinalysis were suspect for tubular injury. Renal biopsy showed calcium oxalate depositions. Elevated plasma and urine oxalate levels established the diagnosis oxalate nephropathy.

The abdominal complaints with steatorrhea and positive anti-tissue transglutaminase antibodies were diagnosed as celiac disease, which was confirmed after duodenal biopsies.

Treatment with prednisone, and gluten-free, low oxalate and normal calcium diet, lowered the plasma oxalate levels and improved his renal function.

Decreased absorption of free fatty acids can lead to increased free oxalate in the colon due to the binding of free fatty acids to calcium, preventing the formation of the less absorbable calcium oxalate in the colon.

Oxalate dispositions in the kidney can lead to acute tubular injury and chronic renal insufficiency. Celiac disease is therefore one of the intestinal diseases that can lead to hyperoxaluria and oxalate nephropathy.

## Introduction

The exact prevalence of oxalate nephropathy is unknown, but it is considered a relatively rare condition and is a potentially underrecognized cause of renal failure [1, 2]. The deposition of oxalate crystals in the tubules and interstitium can result in tubular injury and loss of kidney function [3]. Hyperoxaluria can be primary, due to congenital defects in metabolism of glyoxylate and oxalate [4] or secondary caused by increased absorption, increased intake or decreased intestinal oxalate degradation [3, 5]. Due to increased oxalate uptake in the digestive system, different intestinal diseases are known risk factors for development of nephrolithiasis and nephrocalcinosis.

We present a case of oxalate nephropathy caused by secondary hyperoxaluria in a 72 years old patient with newly diagnosed celiac disease, without nephrolithiasis.

## Case report

A 72-year-old Caucasian male, with a past medical history of eczema, for which he was recently treated with methotrexate, presented with a 3 month history of abdominal discomfort and diarrhea with 5 kg weight loss and fatigue. Colonoscopy in a different medical center did not show any pathology in colon and distal ileum. He had discontinued the methotrexate he used for eczema three months ago because of these abdominal complaints, without clinical improvement. At presentation in our hospital he did not have a fever (99.3 °F (37.4 °C)), mild hypertension (167/83 mmHg) and a normal heart rate (62/min).

He did not use any medication at the time of presentation.

Laboratory tests showed acute renal failure with a creatinine of 290 µmol/L with a normal anion gap metabolic acidosis and no proteinuria (Table 1). Five months prior to presentation his creatinine was 102 µmol/L. C-reactive peptide was 19 mg/L, with leukocytes of 7.2 × 10^9/L. The electrolytes were within normal range. Plasma phosphate during the admission was 1.01 mmol/L. The urine dipstick was negative for erythrocyturia or leucocyturia. Renal ultrasound did not show evidence of hydronephrosis. The kidneys were of normal size and there was no evidence of nephrocalcinosis.

**Table 1:** Laboratory results on the day of admission. MCV: mean corporal volume, eGFR: estimated glomerular filtration rate, LD: Lactate dehydrogenase, AST: Aspartate transaminase, ALT: Alanine transaminase, AP: Alkaline phosphatase: gamma-glutamyl transferase, BE: Base Excess

Parameter	Value	Normal range
White blood cells (×10^9^/L)	7.2	4.0–10.0
Hemoglobin (mmol/L)	6.8	8.5–11.0
Hematocrit (L/L)	0.33	0.40–0.50
MCV (fL)	96	80.0-100.0
Platelet count (×10^9^/L)	291	150–400
C-reactive protein (mg/L)	19	< 5
Glucose (mmol/L)	5.3	4.4–5.5
Sodium (mmol/L)	137	135–145
Potassium (mmol/L)	4.8	3.5-5.0
Chloride (mmol/L)	108	97–107
Creatinine (µmol/L)	290	50–110
eGFR (ml/min*1.73m^2^)	18	45–92
Urea (mmol/L)	16.8	2.5–7.5
Calcium (mmol/L)	2.05	2.20–2.60
Albumin (g/L)	42	35–50
Creatine Kinase (U/L)	167	< 171
LD (U/L)	299	< 248
AST (U/L)	37	< 35
ALT (U/L)	28	< 45
AP (U/L)	98	< 115
GGT (U/L)	18	< 55
Bilirubin, total (mmol/L)	6	< 17
Lactate (mmol/L)	1.0	< 1.0
pH	7.26	7.31–7.41
pCO_2_ (kPa)	4.9	5.5–6.8
HCO_3_^−^ (mmol/L)	17	23–29
BE (mmol/L)	-9.5	-2-+2
Urinalysis		
pH	5.0	4.6-8.0
Leucocytes	Negative	Negative
Nitrate	Negative	Negative
Haem	Negative	Negative
Protein	Negative	Negative
Glucose	Negative	Negative
Ketones	Negative	Negative
Creatinine (mmol/L)	4.0	
Sodium (mmol/L)	37	

He was admitted for further evaluation of renal impairment and abdominal complaints.

Fluid resuscitation did not improve the renal function, which made dehydration due to diarrhea as a cause of renal impairment unlikely. Microscopic urinalysis showed leucocyturia of approximately 10 leukocytes per high-power field, with possible leucocyte casts, and no evidence of glomerular erythrocyturia, consistent with tubular injury. Oxalate crystals were not seen. Remarkably, the initial screening with urine dipstick seems to be falsely negative for leucocyturia.

A renal biopsy was done because the renal function did not improve after rehydration and normalization of defecation pattern.

**Fig. 1:**
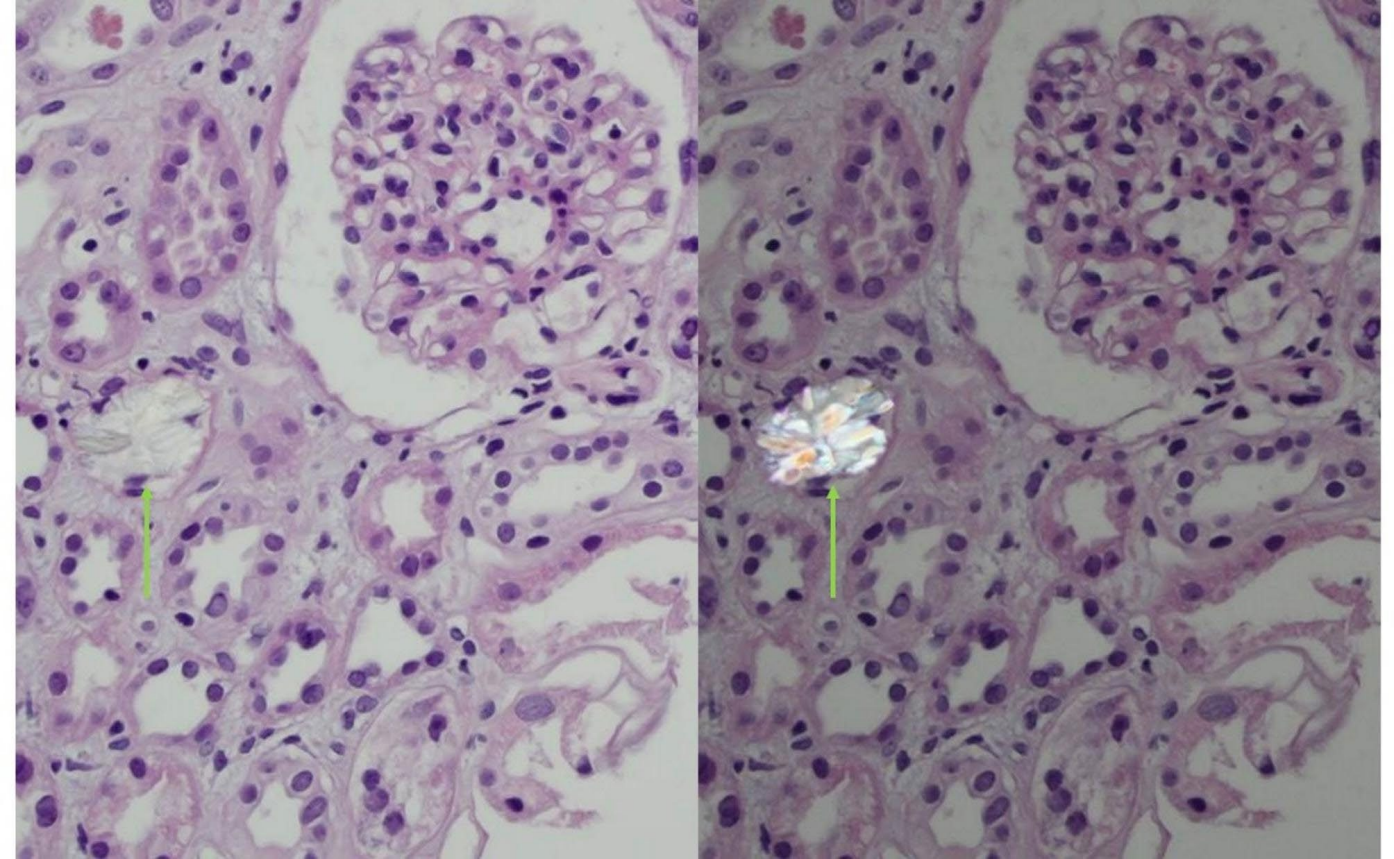
Renal biopsy findings containing calcium oxalate crystals in a tubule (green arrow), with crystals being birefringent under polarized light. There is minimal infiltration around the affected tubule, with degenerative changes. The glomerulus in this section is still intact

3 of 13 glomeruli were globally sclerosed and did not show any abnormalities otherwise. The tubulointerstitium contained multiple zones with atrophy, and fibrosis was present in approximately 20% of the cortical area. Various tubules contained calcium oxalate crystals and showed patchy tubulo-interstitial nephritis with subtle lymphocytic infiltrates (Fig. 1).

Because tubulointerstitial nephritis (TIN), without known causing agent, likely contributed to the renal failure prednisone 30 mg once daily was initiated after the renal biopsy with results pending.

Meanwhile, the abdominal pain was found to be primarily after ingestion of gluten-rich diets, the anti-tissue transglutaminase antibodies were positive (42 U/ml) and there was documented steatorrhea (13.6 g of total fecal fat per day). It was therefore thought to be caused by celiac disease. He was treated with a gluten-free diet, with improvement of the abdominal pain and normalization of fecal pattern.

In the outpatient setting the diagnosis was confirmed after duodenal biopsies showed partial villous atrophy and increased intraepithelial lymphocytes, consistent with celiac disease (Marsh 3a).

Gas chromatography analysis of plasma and urine showed a marked increase in plasma oxalate concentration (28.2 µmol/L [N 2.52–7.03 µmol/L]) and urinary oxalate excretion (1.23 mmol/24 hr [N 0.07–0.72 mmol/24 hr] and 117.23 mmol/mol creatinine [N 8.3–49.00 mmol/mol creatinine]). In combination with low urine citrate, low urinary glycolic acid and glyceric acid this is consistent with secondary hyperoxaluria (Table 2). The combination of calcium oxalate crystals, secondary hyperoxaluria and newly diagnosed celiac disease made oxalate nephropathy in celiac disease the most likely diagnosis. It was treated with a gluten-free, low oxalate and normal calcium diet and high-volume intake. Because of the initial improvement the prednisone was continued.

**Table 2:** Clinical renal function and oxalate levels at presentation and during outpatient follow-up

	At time of diagnosis	8 weeks after presentation	4 months after presentation	6 months after presentation
Plasma creatinine (µmol/L)	230	152	178	179
Creatinine clearance (ml/min)	33	50	52	48
Plasma oxalate (µmol/l)	28.2	8.8	6.9	9.1
24 h-urine oxalate (mmol/mol creatinine)	117.23	67.10	41.97	51.99
24 h-urine oxalate (mmol/24hr)	1.23	0.83	0.56	0.63
24 h-urine glycolate (mmol/mol creatinine)	12.64	38.72	18.54	21.32
24 h-urine glycolate (mmol/24 h)	0.13	0.48	0.25	0.26
24 h-urine glyceric acid (µmol/mol creatinine)	0.62	1.37	0.46	0.55
24 h-urine glyceric acid (µmol/24 h)	6.55	17.04	6.15	6.65
24 h-urine citrate (mmol/mol creatinine)	0.02	0.13	0.10	0.08
24 h-urine citrate (mmol/24 h)	0.21	1.62	1.34	1.00

There was a remarkable improvement of renal function, as well as oxalate levels in plasma and urinary oxalate excretion. The prednisone was discontinued. The patient’s renal function did improve, yet stabilized at a creatinine of 150 µmol/L (Table 2).

## Discussion

First presentation of celiac disease in elderly people is more common than usually expected. Patel et al. showed that 15% of the patients with newly diagnosed celiac were older than 65 years old [6]. The prevalence in a Finnish study showed that approximately 2% of patients between 52 and 74 have biopsy proven celiac disease, which is higher than in the general population [7].

There are few case reports describing methotrexate induced sprue-like syndrome with villous atrophy after initiation of methotrexate [8]. Because in this patient the anti-tissue transglutaminase antibodies were positive and because there was no mucosal healing four months after discontinuation of methotrexate, this was unlikely.

Secondary hyperoxaluria has various causes [5]. The most important etiologies are increased intake of oxalate precursors, for example ascorbic acid supplementation, and increased oxalate availability in the colon due to fat malabsorption in various intestinal diseases.

Even though intestinal diseases, like inflammatory bowel disease [9, 10] and bariatric surgery [11] are known risk factors for urinary stone disease and nephrocalcinosis, and even though celiac disease has been associated with secondary hyperoxaluria and an increased risk of nephrolithiasis [12, 13], case reports of acute renal failure caused by oxalate nephropathy in new onset celiac disease in a patient with previously normal renal function and without nephrolithiasis are rare.

Kohler et al. described a case of new onset celiac disease resulting in oxalate nephropathy [14] and Capolongo et al. described oxalate nephropathy in a patient with subclinical celiac disease, developing after a kidney transplantation [15].

Intestinal diseases with fat malabsorption (e.g. pancreatic insufficiency [16], small bowel resection [11] or inflammatory bowel disease [9, 10]) can lead to increased plasma oxalate levels [17]. Fat malabsorption leads to an increase of free fatty acids in the intestinal lumen. The free fatty acids bind to calcium, reducing the availability of calcium to bind to dietary oxalate. In normal functionality of the intestines, the intestinal absorption of calcium oxalate is less efficient than absorption of free oxalate. The increased concentration of free oxalate in the colon therefore leads to higher plasma concentration. Additionally, free fatty acids and bile salts also increase the permeability of the colon for oxalate, contributing to increased oxalate absorption [18].

Increased plasma oxalate concentration can lead to nephrolithiasis with calcium oxalate stones or the oxalate crystals can precipitate in the renal interstitium and tubules leading to tubular injury and interstitial inflammation, causing oxalate nephropathy.

Moreover, the presence of hypocitraturia, presumably caused by malabsorption [19], facilitates the formation of oxalate crystals, due to impaired complexing of calcium in a soluble form [20].

Lumlertgul et al. [5] performed a systematic review of case reports and case series to examine the clinical characteristics and outcomes of patients with secondary oxalate nephropathy, including different renal biopsy findings. They found that 71% (95% CI: 44–89) of patients had acute tubular injury. Tubular damage and atrophy were found in 69% (95% CI: 43–87). 72% (95% CI 45.0–89.0) showed interstitial mononuclear cell infiltration, and glomerular changes were described in 59% (95% CI: 40–76) of which mostly mesangial cellular proliferation.

There are no randomized controlled trials on therapy for oxalate nephropathy. High volume intake, normal calcium and low oxalate diet, and if possible, treatment for the underlying cause of increased oxalate absorption are important strategies.

Oxalate nephropathy in general has a poor prognosis with studies showing 58% of patients requiring dialysis and only partial, if any recovery of renal function [5].

In this case the fast improvement of renal function cannot be attributed to dissolving of oxalate crystals in the kidney. Most likely the oxalate crystals caused interstitial inflammation, which contributed to the decline in kidney function. This could explain the positive effect of prednisone and the initial increase in renal function. Incomplete recovery may be due to the remaining oxalate crystal deposits in the kidney. Possibly it is comparable to intratubular casts in multiple myeloma, which also results in interstitial inflammation with diffuse fibrosis and tubular atrophy, in which the casts dissolve in weeks [21].

## Conclusion

Oxalate nephropathy is a potentially underrecognized cause of acute renal failure. In various intestinal diseases, fat malabsorption can result in hyperoxaluria, leading to calcium oxalate depositions in the kidneys, causing renal insufficiency. Even though there are numerous reports on oxalate nephropathy in intestinal disease, new onset celiac disease as a cause of oxalate nephropathy has been rarely recognized.

Because celiac disease is more common later in life than what is usually assumed, in patients in any age group with a combination of known celiac disease or clinical suspicion, and acute renal failure, physicians should be aware of oxalate nephropathy secondary to celiac disease.

## Data Availability

Not applicable

## Abbreviations

TIN: Tubulointerstitial nephritis
